# A systematic review of self-esteem and related factors among burns patients

**DOI:** 10.1016/j.amsu.2022.104811

**Published:** 2022-11-08

**Authors:** Alireza Mehrabi, Atefeh Falakdami, Amirabbas Mollaei, Poorya Takasi, Pooyan Ghorbani Vajargah, Hedayat Jafari, Seyyed Mohammad Hossein Mazloum, Negin Rahimzadeh, Mohammad Javad Ghazanfari, Amir Emami Zeydi, Mohammadreza Mobayen, Samad Karkhah

**Affiliations:** a Department of Psychology, School of Medicine, Guilan University of Medical Sciences, Rasht, Iran; b Department of Medical-Surgical Nursing, School of Nursing and Midwifery, Guilan University of Medical Sciences, Rasht, Iran; c Associate Professor of Nursing, Department of Medical Surgical Nursing, School of Nursing and Midwifery, Traditional and Complementary Medicine Research Center, Addiction Institute, Mazandaran University of Medical Sciences, Sari, Iran; d Burn and Regenerative Medicine Research Center, Guilan University of Medical Sciences, Rasht, Iran; e Department of Medical-Surgical Nursing, School of Nursing and Midwifery, Shahid Beheshti University of Medical Sciences, Tehran, Iran; f Department of Medical-Surgical Nursing, Nasibeh School of Nursing and Midwifery, Mazandaran University of Medical Sciences, Sari, Iran

**Keywords:** Burns, Self-esteem, Self-concept, Systematic review

## Abstract

**Introduction:**

The present systematic review was conducted to examine self-esteem and related factors in burns patients.

**Methods:**

A comprehensive search was conducted from the first to the April 1, 2022 at the international electronic databases such as Scopus, PubMed, Web of Science, and Persian electronic databases such as Iranmedex, and Scientific Information Database using keywords extracted from Medical Subject Headings such as "Burns", "Self-confidence", "Self-perception", "Self-esteem", and "Self-concept".

**Results:**

A total of 762 burn patients were included in this review from ten cross-sectional studies. The mean score of self-esteem in burn patients based on Rosenberg Self-Esteem Scale, State Self-Esteem Scale, Cooper Smith's self-esteem questionnaire, and Rifai's self-esteem scale were 17.77 (SD = 5.55) out of 30, 65.91 (SD = 5.49) out of 100, 56.68 (SD = 5.49) out of 100, and 109.77 (SD = 9.55) out of 145, respectively. Factors associated with self-esteem in burns patients were gender, occupation, the location of the burn, type of burn, site of burn, burn scar, and quality of life had a significant relationship with burn patients' self-esteem. Factors such as social support, family support, friends support, and supporting others, had a significant positive relationship with self-esteem of burns patient. However, self-esteem in patients with burn had a significant negative relationship with grade of burn injury, percentage of burn, depth of burn, facial burn, post-traumatic stress disorder, psychiatric morbidity, major depressive, and suicidality factors.

**Conclusion:**

Overall, patients with burns had moderate levels of self-esteem. Therefore, it is recommended that health professionals use interdisciplinary approaches to better manage burn patients.

## Highlights

• Patients with burns had moderate levels of self-esteem.
• Gender, occupation, the location of the burn, type of burn, site of burn, burn scar, and quality of life had a significant relationship with burn patients' self-esteem.
• Social support, family support, friends support, and supporting others had a significant positive relationship with self-esteem of burns patients.
• Bruns grade, burn depth, facial burn, post-traumatic stress disorder, psychiatric morbidity, major depressive, and suicidality factors had a significant negative relationship with burn patients' self-esteem.

## 1 Introduction

Burn injuries, as a major public health problem, can lead to high morbidity and mortality [[1], [2], [3], [4], [5], [6], [7], [8], [9], [10], [11], [12], [13], [14], [15], [16], [17], [18], [19], [20], [21], [22]]. Burn injuries occur following direct contact with surfaces, fire, hot liquids, chemicals, gases, electricity, or radiation, which cause tissue damage inside or outside the body [23]. According to the latest statistics from the World Health Organization, burn injuries are the fourth most common traumatic injury, accounting for 180,000 deaths annually and its incidence is high in low- and middle-income countries [24].

Burns as a destructive injury can be associated with a wide range of complications [25]. In other words, physical injuries due to burns such as changes in appearance, scar formation, especially in the face, and loss of an organ can lead to many psychological and social complications and lead to changes in the quality of life of these patients [26]. Due to its impact on quality of life, it is important to pay attention to its most significant dimension, which is self-esteem [27]. From Rosenberg's point of view, self-esteem means, “a sense of self-sufficiency to face the fundamental challenges of life and to be worthy of happi-selectiveness”. Rosenberg believes that self-esteem is the difference between the perceived self and the ideal self, which decreases with increasing differences between these two components [28,29]. In other words, self-esteem determines a negative or positive attitude towards various aspects of life and if it is damaged, it will be difficult and impossible for these people to endure difficult living conditions [30]. The growth of self-esteem begins at birth and changes throughout life due to the influence of various experiences and factors [30]. Burn patients are no exception to this rule and their self-esteem can be related to various factors such as demographic, clinical, and psychological [29,31,32].

Due to advances in the treatment of burn patients, patient survival has increased. However, burns can cause complications and problems in a person's appearance that may last a lifetime and can eventually lead to social and psychological problems such as low self-esteem [33]. Less attention is paid to the emotional needs of burn patients than to their survival and physical needs [34]. As a result, low self-esteem has a negative effect on interpersonal relationships, focus, feeling, and performance of burn patients [29]. Therefore, due to the importance of this issue and the lack of a review study in this field, the present review study was conducted to examine self-esteem and related factors in burns patients.

## 2 Methods

This review were conducted based on the Preferred Reporting Items for Systematic Reviews and Meta-Analysis (PRISMA) checklist (Supplementary Table S1) [35]. This systematic review is not registered in the international prospective register of systematic reviews (PROSPERO) database.

### 2.1 Search strategy

A comprehensive search was conducted from the first to the April 1, 2022 at the international electronic databases such as Scopus, PubMed, Web of Science, and Persian electronic databases such as Iranmedex, and Scientific Information Database (SID) using keywords extracted from Medical Subject Headings such as "Burns", "Self-confidence", "Self-perception", "self-esteem", and "Self-concept". For example, the search strategy was in PubMed/MEDLINE database including ((“Self-confidence”) OR (“Self-perception”) OR (“Self-perception”) OR (“self-esteem”) OR (“Self-concept”)) AND ((“Burns”) OR (“Burns patients”)). The keywords were combined using the Boolean operators "AND" and "OR". A search was conducted in Iranian electronic databases using the mentioned keywords in Persian. The search process was completed separately by two researchers. Gray literature articles such as expert opinions, conference presentations, dissertations, research and committee reports, and ongoing research were not included in this review articles. Gray literature includes articles published in print and electronic formats but not evaluated by a commercial publisher [36].

### 2.2 Inclusion and exclusion criteria

In this systematic review, cross-sectional studies published in English and Persian according to the subject of self-esteem and related factors in burn patients were included. Letters to the editor, case reports, conference proceedings, experiments, studies with qualitative designs, and reviews were excluded.

### 2.3 Study selection

The data in this review was managed using EndNote X8 software. Based on the inclusion and exclusion criteria, two researchers separately evaluated the study selection criteria, which included the elimination of duplicate studies, evaluation of the title and abstract of the study, and evaluation of the full text of the articles, first electronically and then manually. The third researcher resolved the differences between the first two evaluators regarding the evaluation of the studies. Finally, the study reference list was manually evaluated to prevent data loss.

### 2.4 Data extraction and quality assessment

Researchers extracted information including the name of the first author, year of publication, location, sample size, male/female ratio, age, single/married ratio, level of education, occupation, residence of area, type of burn injury, grade of burn injury, site of the burn, questionnaire, and key results. The quality of the included studies in this review was assessed by using the appraisal tool for cross-sectional studies (AXIS tool). This tool evaluates the quality of the included studies via 20 items with a two-point Likert, including yes (score of 1) and no (score of 0). This tool assesses report quality (7 items), study design quality (7 items), and the possible introduction of biases (6 items). Finally, AXIS rates the quality of studies at three levels: high (70–100%), fair (60–69.9%), and low (0–59.9%) [37]. Two researchers conducted data extraction and qualitative evaluation of the studies separately. Also, the AMSTAR 2 checklist was completed to evaluate the study quality (Supplementary File S2) [38].

## 3 Results

### 3.1 Study selection

**Fig. 1:**
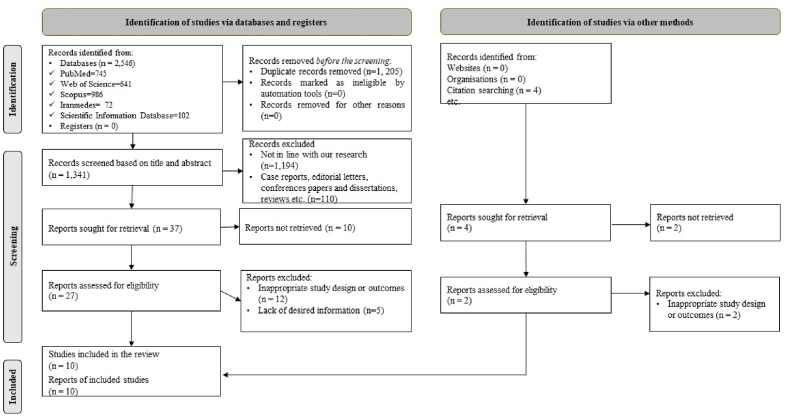
Flow diagram of the study selection process.

According to Fig. 1, in this systematic review, 2546 studies were obtained by searching electronic databases. 1341 The article remained after the elimination of duplicate studies. 1189 studies were deleted after a thorough review of the title and abstract of the articles due to inconsistencies with the purpose of this review. Also, 107 articles were deleted due to being non-cross-sectional. Twelve studies were deleted due to poor design or study results after a comprehensive review of the text of the articles. Five studies were also omitted due to a lack of appropriate information. Finally, ten studies [29,31,32,[39], [40], [41], [42], [43], [44], [45]] were included in this systematic review.

### 3.2 Study characteristics

A total of 762 burn patients were included in this review from ten cross-sectional studies [29,31,32,[39], [40], [41], [42], [43], [44], [45]]. 54.30% and 61.71% of burn patients were female and married, respectively. The mean age of burn patients was 32.07 (SD = 9.96). 34.47% of burn patients were unemployed and 25.60% were employed (n = 5) [29,40,42,44,45]. 50.73% of burn patients suffered from thermal burns (n = 7) [29,32,39,40,[42], [43], [44]]. 44.87% of patients had third grade burns (n = 4) [32,41,43,44]. Six studies also reported the site of the burn [29,32,40,41,44,45]. To assess self-esteem in burn patients, six studies [29,31,[41], [42], [43], [44]] used the Rosenberg Self-Esteem Scale (RSES), two studies [40,45] used the State Self-Esteem Scale (SSES), one study [39] used Cooper Smith's self-esteem questionnaire, and one study [32] used Rifai's self-esteem scale. Of the studies included in this systematic review, four [32,40,43,45] were conducted in Pakistan, three [31,41,42] in India, two [29,39] in Iran, and one [44] in Egypt. Table 1 presents the characteristics of the included articles.

**Table 1:** Basic characteristics of the included studies in this systematic review.

First Author/year	Location	Sample size	M/F ratio (%)	Age (mean ± SD)	Single/Married ratio (%)	Level of education (lower intermediate and intermediate/upper-intermediate)	Occupation (%)	Residence of area (urban/rural) (%)	Type of burn injury (%)	Grade of burn injury (%)	Site of the burn (%)	Questionnaire	Key results	AXIS Score
Enayati et al.*,* 2006 [39]	Iran	60	48.33/51.67	28.53 (SD = 11.02)	48.33/51.67	N/A	N/A	N/A	Chemical	N/A	N/A	Cooper Smith's self-esteem questionnaire	• The mean score of self-esteem was 56.68 (SD = 5.49).	Fair
Faisal et al.*,* 2016	Pakistan	100	53.00/47.00	N/A	5.00/95.00	96.00/4.00	• Unemployed (38.00)	N/A	• Thermal (5.00)	N/A	Facial	SSES	• The mean score of self-esteem was 56.94 (SD = 7.38).	
[40]							• Employed (37.00)		• Chemical (22.00)				• 66.00% of patients had a below-average score in self-esteem scores.	High
							• Self-employed (4.00)		• Industrial (64.00)				• There was a significant relationship between facial burns and low self-esteem (P = 0.001).	
							• Housewife (21.00)		• Electrical (9.00)				• Women had higher self-esteem scores than men (P = 0.001).	
													• Housewives had higher self-esteem scores than others (P = 0.001).	
													• Patients with chemical burns had higher self-esteem scores than others (P = 0.001)	
													• Patients with incidental burns had higher self-esteem scores than accidental (P = 0.001).	
													• Patients who had sustained the injury at home had a higher self-esteem score than those who had sustained the injury in the workplace (P = 0.001).	
Jain et al.*,* 2017	India	100	54.00/46.00	34.15 (SD = 10.80)	31.00/69.00	70.00/30.00	N/A	N/A	N/A	• First (46.00)	• Facial (57.00)	RSES	• 71.74% of patients with first-grade burn, had normal self-esteem.	High
[41]										• Second (37.00)	• Others (43.00)		• 64.86% of patients with second-grade burn, had normal self-esteem.	
										• Third (17.00)			• 58.82% of patients with third-grade burn, had normal self-esteem.	
Zahid et al.*,* 2017	Pakistan	100	53.00/47.00	N/A	5.00/95.00	96.00/4.00	• Unemployed (38.00)	N/A	N/A	N/A	Facial	SSES	• The mean score of self-esteem was 74.88 (SD = 6.90).	High
[45]							• Employed (37.00)						• Male patients had a higher score of self-esteem than female patients (t = 6.226, P < 0.05).	
							• Self-employed (4.00)							
							• Housewife (21.00)							
Zaidi et al.*,* 2017	Pakistan	40	62.50/37.50	28.28 (SD = 4.60)	50.00/50.00	20.00/80.00	N/A	N/A	• Thermal (67.50)	Third (100)	• Face and chest (17.50)	Rifais' self-esteem scale	The mean score of self-esteem was 109.77 (SD = 9.55).	High
[32]									• Hot water (32.50)		• Hand and chest (57.50)			
											• Different body parts (25.00)			
Mujeeb &	Pakistan	62	0/100	29.34 (SD = 9.18)	48.39/51.61	95.16/4.84	N/A	N/A	• Chemical (45.17)	• First (30.65)	N/A	RSES	• The mean score of self-esteem was 21.30 (SD = 3.76).	High
Tariq, 2019									• Thermal (35.48)	• Second(50.00)			• Minor burns patients had a higher score of self-esteem than severe burns patients (P < 0.01).	
[43]									• Explosive (19.35)	• Third(19.35)			• There was a significant negative relationship between self-esteem and PTSD (β = −0.399, P = 0.001).	
														
Saad, 2019 [44]	Egypt	50	50.00/50.00	36.32 (SD = 13.12)	36.00/64.00	88.00/12.00	• Worker (44.00)	48.00/52.00	• Thermal (50.00)	• Second (60.00)	• Face	RSES	• 40.00% of patients had low self-esteem (<15) in the first week of burns.	High
							• Housewife (40.00)		• Hot water (36.00)	• Third (40.00)	• Trunk		• The mean score of self-esteem in the first week of burns was 13.00 (SD = 7.80).	
							• Employed (10.00)		• Chemical (8.00)		• buttocks		• 88.00% of patients had low self-esteem (<15) in the eight weeks of burns.	
							• Unemployed (6.00)		• Electrical (6.00)		• Thighs		• The mean score of self-esteem in the eighth week of burns was 9.24 (SD = 3.92).	
											• Legs		• There was a significant negative relationship between self-esteem and the percentage of burn (P = 0.014).	
											• Feet		• There was a significant negative relationship between self-esteem and grade of burn (p = 0.002).	
											• Others		• There was a significant negative relationship between self-esteem and depth of burn (P < 0.05).	
													• There was a significant relationship between self-esteem and sites of burns (P < 0.05).	
													• There was a significant relationship between self-esteem and burn scar (P < 0.05).	
Selvamani et al.*,* 2019 [31]	India	30	50.00/50.00	35.00 (SD=N/A)	26.67/73.33	N/A	N/A	73.33/26.67	N/A	N/A	N/A	RSES	The mean score of self-esteem in burn patients was 18.07 (SD=N/A).	High
Ghorbani et al.*,* 2020 [29]	Iran	120	39.17/60.83	35.40 (SD = 12.38)	37.50/62.50	80.00/20.00	• Housewife (32.50)	N/A	• Thermal (69.16)	N/A	• Face (0.83)	RSES	• The mean score of self-esteem was 2.70 (SD = 4.95).	High
							• Unemployed (8.33)		• Hot water (15.00)		• Upper limb (0.83)		• There was a significant positive relationship between social support and self-esteem (p = 0.001, r = 0.288).	
							• Worker (12.50)		• Electrical (4.18)		• Lower limbs (3.33)		• There was a significant positive relationship between family support and self-esteem (p = 0.006, r = 0.25).	
							• Employed (20.00)		• Chemical (11.66)		• Face and upper limb (11.68)		• There was a significant positive relationship between friends' support and self-esteem (p = 0.033, r = 0.195).	
							• Self-employed (23.33)				• Face and lower limb (0.83)		• There was a significant positive relationship between supporting others and self-esteem (p = 0.001, r = 0.289).	
							• Student (3.34)				• Face and trunk (2.50)			
											• Upper and lower limb (7.50)			
											• Upper and trunk (3.33)			
											• More than three areas (69.17)			
Kadam et al.*,* 2021 [42]	India	100	47.00/53.00	29.56 (SD = 8.64)	25.00/75.00	95.00/5.00	• Unemployed (72.00)	N/A	• Thermal (62.00)	N/A	N/A	RSES	• The mean score of self-esteem was 18.70 (SD = 5.08).	High
							• Employed (24.00)		• Hot water (34.00)				• There was a significant negative relationship between psychiatric morbidity and self-esteem (P < 0.00001).	
							• Student (4.00)		• Chemical (4.00)				• There was a significant negative relationship between major depressive disorder and self-esteem (P < 0.00001).	
													• There was a significant negative relationship between suicidality and self-esteem (P < 0.00001).	
													• There was a significant relationship between quality of life and self-esteem (P < 0.00001).	

**RSES:** Rosenberg Self-Esteem Scale; **SSES:** State Self-Esteem Scale.

### 3.3 Methodological quality of included study

**Fig. 2:**
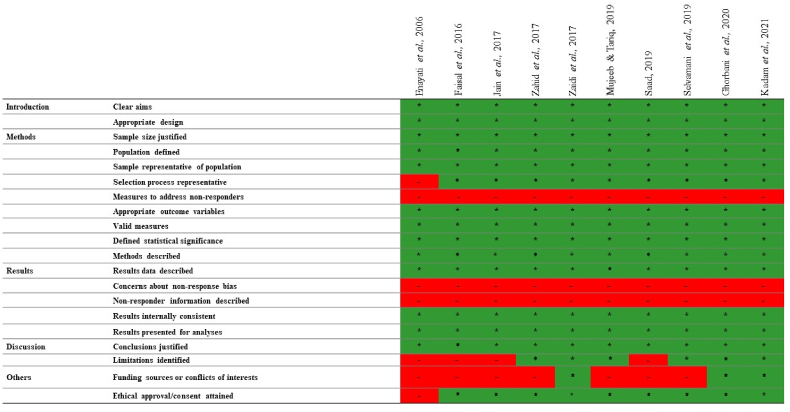
Assessment of the quality of the included articles.

Of the ten studies [29,31,32,[39], [40], [41], [42], [43], [44], [45]] included in this review, nine [29,31,32,[40], [41], [42], [43], [44], [45]] had high-quality studies and one [39] fair quality study (Fig. 2).

### 3.4 Self-esteem in burn patients

The mean score of self-esteem in burn patients based on RSES [29,31,[41], [42], [43], [44]], SSES [40,45], Cooper Smith's self-esteem questionnaire [39], and Rifai's self-esteem scale [32] were 17.77 (SD = 5.55) out of 30, 65.91 (SD = 5.49) out of 100, 56.68 (SD = 5.49) out of 100, and 109.77 (SD = 9.55) out of 145, respectively. Overall, patients with burns had moderate levels of self-esteem.

### 3.5 Factors associated with the burn's patients' self-steam

Factors associated with self-esteem in burns patients were gender (n = 2) [40,45], occupation (n = 2) [40,45], the location of the burn (n = 2) [40,45], type of burn (n = 2) [40,45], site of burn (n = 1) [44], burn scar (n = 1) [44], and quality of life (n = 1) [42] had a significant relationship with burn patients self-esteem. Factors such as social support (n = 1) [29], family support (n = 1) [29], friends support (n = 1) [29], and supporting others (n = 1) [29], had a significant positive relationship with self-esteem of burns patient. However, self-esteem in patients with burn had a significant negative relationship with grade of burn injury (n = 3) [41,43,44], percentage of burn (n = 1) [44], depth of burn (n = 1) [44], facial burn (n = 1) [40], post-traumatic stress disorder (n = 1) [43], psychiatric morbidity (n = 1) [42], major depressive (n = 1) [42], and suicidality (n = 1) [42] factors.

## 4 Discussion

This systematic review showed that patients with burns had moderate levels of self-esteem. Factors associated with self-esteem in burns patients were gender, occupation, the location of the burn, type of burn, site of burn, burn scar, and quality of life had a significant relationship with burn patients’ self-esteem. Factors such as social support, family support, friends support, and supporting others, had a significant positive relationship with self-esteem of burns patient. However, self-esteem in patients with burn had a significant negative relationship with grade of burn injury, percentage of burn, depth of burn, facial burn, post-traumatic stress disorder, psychiatric morbidity, major depressive, and suicidality factors.

Burn injuries are a major health problem in the world that affects the mental, social and physical condition of patients [46]. In the burn process, the patient's self-esteem is affected and leads to a negative attitude towards the existing conditions; therefore, it is important to pay attention to this issue [47]. The results of the present systematic review showed that burn patients have moderate self-esteem. However, the difference in self-esteem of burn patients can be due to the influence of factors such as demographic characteristics, burn characteristics, social support, psychiatric complications, depression, post-traumatic stress disorder, quality of life, and suicide.

Based on the results of this study, quality of life is an influential factor in patients' self-esteem. In this regard, a study in Pakistan showed that the quality of life of most burn patients has deteriorated after injury [48]. Also, another study reported a significant relationship between burn severity and patients' quality of life, as the burn increases, the quality of life of patients decreases [49]. Therefore, health managers and policymakers must identify the various dimensions of burns and improve burn management and patient care to improve quality of life.

As presented in this study, posttraumatic stress disorder and perceived social support had a significant relationship with patients’ self-esteem. A study in Pakistan showed that burn patients, had high post-traumatic stress and low social support [50]. The results of another study showed that the rate of depression in burn patients was a significant relationship with the severity of burns. Showed that, depression increases with the severity of burns [51]. A review study found that burn survivors had higher suicidal ideation and a higher suicide rate than the general population [52]. Therefore, the clinical management of burn patients should be based on an interdisciplinary approach to ensure the high quality of clinical, psychological, and social care. Based on the findings of the present systematic review, it is suggested that future studies apply educational strategies to prevent psychosocial complications of burns with a focus on self-esteem.

### 4.1 Limitations

The present systematic review had some limitations that should be noted. Due to the methodological diversity and different tools, the conduct of meta-analysis was not possible in this study. The absence of meta-analysis in the study increases the heterogeneity of the findings, but the systematic approach to data collection, sorting, and analysis of studies in the present systematic review study remained strong enough. Not all studies in this field may be found, despite a thorough search of all electronic databases. Finally, in this systematic review, only studies in English and Persian were included, so articles in other languages may not be included in this study.

### 4.2 Implications for health managers and policymakers

Decreased self-esteem is one of the most important issues in burn patients because it can cause a negative outlook on patients' lives and reduce their quality of life. Health policymakers and managers can prevent psychosocial problems, especially low self-esteem, by applying educational strategies. Also, clinical management in burn patients should be based on an interdisciplinary approach among health professionals in various psychosocial and clinical areas to provide comprehensive care for these patients.

### 4.3 Implication for future research

Based on the results of this systematic review, it is suggested that in future studies, more studies be conducted on the factors affecting the self-esteem of burn patients. Also, articles in this field have been done in only four countries in Asia, so it is suggested that this issue be examined in other parts of the world. Based on the research data collected, it may be possible to conduct research in the future by adding variables such as the estimated burn area of the patient, the length of stay during treatment, and the patient's constraints when experiencing burns, especially during mobilization.

## 5 Conclusion

In sum, this systematic review showed that patients with burns had moderate levels of self-esteem. Factors associated with self-esteem in burns patients were gender, occupation, the location of the burn, type of burn, site of burn, burn scar, and quality of life had a significant relationship with burn patients’ self-esteem. Factors such as social support, family support, friends support, and supporting others, had a significant positive relationship with self-esteem of burns patient. However, self-esteem in patients with burn had a significant negative relationship with grade of burn injury, percentage of burn, depth of burn, facial burn, post-traumatic stress disorder, psychiatric morbidity, major depressive, and suicidality factors. Therefore, it is recommended that health professionals use interdisciplinary approaches to better manage burn patients.

## Ethical approval

This study is a systematic review and does not require ethical approval and consent.

## Sources of funding

None.

## Author contribution

Study concept and design by all authors; Data acquisition by all authors; Data interpretation by all authors; drafting the manuscript by all authors; Revision of the manuscript by all authors; the final version of the manuscript is approved by all authors.

## Registration of research studies

1.Name of the registry: None
2.Unique Identifying number or registration ID: None
3.Hyperlink to your specific registration (must be publicly accessible and will be checked): None

## Guarantor

Samad Karkhah.

## Consent

This study is a systematic review and does not require ethical approval and consent.

## Data availability

The datasets generated and analyzed during the current study are available from the corresponding author on reason-able request.

## Provenance and peer review

Not commissioned, externally peer-reviewed.

## Declaration of competing interest

None.
